# Influence of Microclimatic Variations on Morphological Traits of Ferns in Urban Forests of Central Veracruz, Mexico

**DOI:** 10.3390/plants14111732

**Published:** 2025-06-05

**Authors:** Jessica G. Landeros-López, Thorsten Krömer, Jorge A. Gómez-Díaz, Noé Velázquez-Rosas, César I. Carvajal-Hernández

**Affiliations:** 1 Centro de Investigaciones Tropicales, Universidad Veracruzana, Xalapa 91000, Mexico; jessgll@hotmail.com (J.G.L.-L.); tkromer@uv.mx (T.K.); novelazquez@uv.mx (N.V.-R.); 2 Instituto de Investigaciones Biológicas, Universidad Veracruzana, Xalapa 91000, Mexico; jorggomez@uv.mx

**Keywords:** plant communities, vegetation structure, pteridophytes, anthropized ecosystems, functional traits, environmental stress

## Abstract

Urban forests are remnants of forest habitats within urban areas. Their structural alterations create stressful microclimatic conditions that can influence the morphology of sensitive plants, such as ferns. This study analyzed variations in the morphological traits of ferns in four urban forest sites in central Veracruz, Mexico, considering the microclimatic differences arising from vegetation structure. Temperature, humidity, canopy openness, and radiation were measured, along with eight foliar traits, while assessing the impact of site and habit (terrestrial or epiphytic) on the response. Sites with greater alterations in vegetation structure exhibited increased canopy openness, solar radiation, temperature, and a higher number of days with lower relative humidity. In these sites, leaves showed an increase in dry matter content and vein density, indicating a greater investment in resource storage and structural resistance. In the less-disturbed sites, terrestrial ferns demonstrated larger leaf area and specific leaf area, suggesting greater growth potential. Conversely, epiphytes generally had smaller leaves, which could represent an adaptive advantage for these species. The results also suggest a process of biotic homogenization within this plant group, reflecting a similar morphological response, except for indicator species restricted to less disturbed sites. Thus, this study reveals that microclimatic variations induced by urbanization significantly affect plant morphology and, ultimately, species diversity.

## 1. Introduction

Urban forests are plant communities with regenerative capacity characterized by a well-defined tree canopy and lower strata. They are formed from remnants of natural habitats or secondary vegetation that arise from natural or induced regeneration, currently surrounded by urban infrastructure [1,2]. These forests exhibit variation in structure and plant species composition, ranging from structurally complex native forest fragments to simplified systems dominated by a few exotic species [3,4,5]. Their ecological importance lies in their contribution to regulating temperature, as well as water, soil, and air flows in urban ecosystems, while also providing habitat for biodiversity [6,7].

Like other ecosystems, these forests are influenced by a wide range of factors, such as climate, substrate, resident organisms, relief, and the history of the system [8]. However, urban forests are also impacted by anthropogenic effects [9]. Proximity to urban environments results in several negative consequences for these areas due to changes in land use, the influence of pollutants, continuous recreational pressures (which impact the environment through soil compaction, species extraction, alteration of fauna, etc.), separation from propagule sources, reduced regeneration capacity, and the introduction of exotic species [2,9]. These factors affect the structure and density of existing vegetation, which, in turn, impacts the physical environment of these forest ecosystems, leading to increased solar radiation, higher temperatures, greater evaporation, and lower relative humidity, along with the heat island effect and heat retention by urban surfaces [10,11,12].

The set of these alterations represents stressful environmental conditions, which induce changes in ecosystem processes and the ecology of the organisms present in these forests [13,14]. In plant species, the selection of more tolerant organisms to the adverse conditions of the urban environment described above has been observed, evidenced by their phenotypic plasticity and traits that enhance resilience [9,15]. At the same time, native and sensitive species are eliminated, which ultimately leads to a reduction in diversity, resulting in a process of biotic homogenization [9,16,17,18].

Ferns are vascular plants that do not depend on any pollination mechanism, since their reproduction is carried out through the dispersion of spores by wind or water [19,20]. Thus, the distribution and establishment of this group allow them to reflect the abiotic conditions of their habitat [21]. Additionally, this group of plants has a close relationship with environmental humidity due to their limited hydraulic capacity and passive stomatal control [22,23]. Many ferns also have a certain dependence on trees, which provide shaded environments and act as hosts for epiphytic species [24,25]. Therefore, it is understood that alterations in light and humidity conditions can significantly affect their development and establishment, making them particularly sensitive to disturbances in their environment [26,27,28,29].

The above mentioned has been demonstrated through a remarkable variation in their functional responses to different environmental conditions [30,31,32]. Specifically, changes at the microenvironmental level can influence variations in their morphological traits [33,34]. For example, conditions of high light intensity and water limitation tend to produce smaller leaves [30,35,36]. Likewise, sites with extreme temperatures, as well as low relative humidity and higher solar radiation, indicate stressful conditions for these organisms, resulting in leaves with greater laminar thickness, moisture content, and dry mass per unit area [31,37,38]. This reflects a protective response to abiotic stressors in this group. However, to our knowledge, there are no studies related to functional or morphological variation in ferns exclusively in urban forests.

The Xalapa–Banderilla conurbation area is situated in central Veracruz, Mexico, originally characterized by humid montane forest (HMF) vegetation and known as the region with the highest diversity of ferns within the state [27,39,40]. However, this area has experienced significant demographic growth in recent decades, leading to a direct transition from woody vegetation to urban usage, resulting in a noticeably fragmented landscape [41]. Currently, the urban vegetation comprises both native and introduced species distributed throughout its network of green areas, among which forest fragments in various stages of anthropization stand out [42,43,44]. These forests provide essential ecosystem services to the capital city of Xalapa, including climate regulation, carbon sequestration, and serving as a refuge for biodiversity [45,46,47]. However, recent studies in the area indicate variations in the structure and composition of the urban forests, reflecting their environmental and anthropogenic usage history [42,47,48]. These differences may influence microclimatic conditions, which directly affect the functional responses and distribution of the present fern communities.

Therefore, the objective of this study was to evaluate the variation in morphological traits of terrestrial and epiphytic ferns most representative of four urban forest sites in central Veracruz, Mexico, in response to microclimatic variation associated with the vegetation structure of these forests. This study adopts a functional ecology approach, focusing on how morphological traits reflect the functional responses of ferns to environmental conditions. We hypothesize that the microclimatic conditions of each urban forest are determined by the vegetation structure of the corresponding forest. These conditions, in turn, will influence the functional response of the ferns, which will be manifested in morphological traits adapted to the microclimatic characteristics of each site. Thus, this evaluation will allow us to better understand some of the adaptive mechanisms of this group of plants in urbanized ecosystems.

## 2. Results

### 2.1. Microclimate

**Figure 1:**
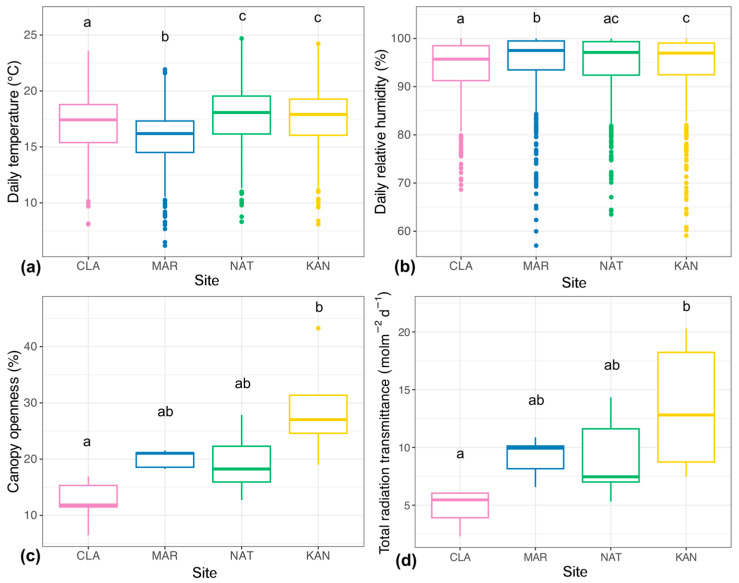
Microclimatic variables measured at the four study sites from August 2021 to August 2023. These include (**a**) daily temperature; (**b**) daily relative humidity; (**c**) canopy openness; and (**d**) total photosynthetic radiation transmittance. CLA: ANP Francisco Javier Clavijero, MAR: ANP La Martinica, NAT: Parque Natura, KAN: Kaná Agroforestry. Different letters above the boxes indicate significant differences between sites (*p* < 0.05).

The evaluated microclimatic variables revealed differences between sites. For temperature (Kruskal–Wallis: H = 244.25, df = 3, *p* < 0.0001), the highest daily mean was recorded at NAT (17.84 °C), while the lowest was found at MAR (15.83 °C; Figure 1a). Sites at approximately the same altitude (CLA, NAT, and KAN) showed a variation of less than 0.5 °C among them, but a difference greater than 1.5 °C in comparison to MAR, the site at the highest elevation (Figure 1a). Regarding relative humidity, variations of less than 2% were observed among the four sites, but with statistical significance (Kruskal–Wallis: H = 41.37, df = 3, *p* < 0.0001; Figure 1b). In this case, MAR recorded the highest mean (95.03%), while CLA had the lowest (93.83%; Figure 1b).

The canopy openness and radiation transmittance variables responded similarly to one another. In this case, KAN exhibited the highest values (29.0% and 13.5 mol/m^2^d, respectively; Figure 1c,d), followed by MAR and NAT, while CLA recorded the lowest values (12.4% and 4.77 mol/m^2^d). This finding was reflected in the statistically significant differences in both variables, according to the ANOVA results (F (3, 16) = 6.81, *p* < 0.01; F (3, 16) = 4.93, *p* < 0.05, respectively), particularly between the CLA and KAN sites (Figure 1c,d).

**Figure 2:**
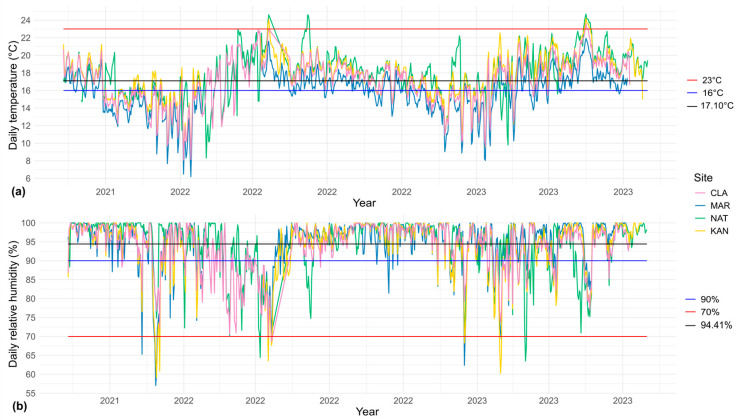
Time series of daily temperature and relative humidity for the four study sites from August 2021 to August 2023. (**a**) Time series of daily temperature means; (**b**) time series of daily relative humidity means. Black lines indicate the mean values across the four sites (temperature: 17.10 °C; relative humidity: 94.41%). Red and blue lines represent environmental thresholds observed in HMF of the region in previous studies [27,49]: red = values typically found in disturbed sites (temperature: 23 °C; relative humidity: 70%), blue = values characteristic of well-conserved sites (temperature: 16 °C; relative humidity: 90%). CLA: Francisco Javier Clavijero, MAR: La Martinica, NAT: Natura Park, KAN: Kaná Agroforest.

The time series analysis confirmed that NAT and KAN were the warmest sites, as they recorded the highest number of days with temperatures exceeding 23 °C and the fewest days below 16 °C (Figure 2a; Supplementary Table S1). In contrast, MAR exhibited the opposite pattern, registering the lowest daily temperatures, with values close to 6 °C (Figure 2a). Moreover, MAR had the highest number of days with temperatures below 16 °C and none above 23 °C (Supplementary Table S1). CLA followed MAR in the number of days below 16 °C and had only a minimal proportion of days exceeding 23 °C (Supplementary Table S1).

Regarding relative humidity, MAR and KAN recorded the highest number of days below 70% (Figure 2b; Supplementary Table S2). Nonetheless, MAR also showed the highest proportion of daily values above the overall mean of the four sites (94.41%; Supplementary Table S2). In the case of NAT, a progressive decrease in relative humidity was observed starting in 2022 (Figure 2b). Meanwhile, CLA showed the least variation over the two-year period, with neither the lowest nor the highest values for temperature or humidity (Figure 2).

### 2.2. Principal Component Analysis

**Figure 3:**
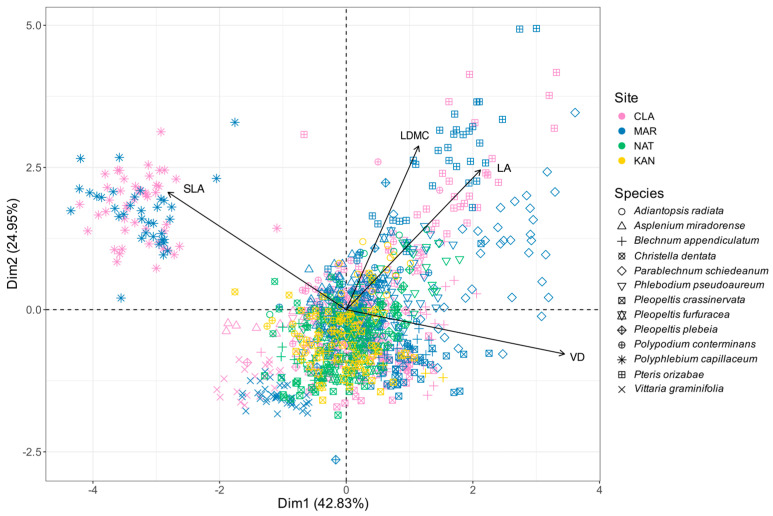
Principal component analysis of the morphological traits of ferns present at the four study sites. CLA: Francisco Javier Clavijero; MAR: La Martinica; NAT: Natura Park; KAN: Kaná Agroforest; LA: leaf area; SLA: specific leaf area; LDMC: leaf dry matter content; VD: vein density.

Principal component analysis (PCA) revealed that the first component (Dim. 1) explained most of the variability with a variance of 42.83%. This was primarily influenced by the variables VD and SLA, contributing 46.41% and 30.94%, respectively, and exhibiting opposite orientations according to their correlations (positive for VD: 0.86, negative for SLA: −0.70; Figure 3). The second component (Dim. 2) accounts for 24.95% of the variance, with the variables LDMC and LA being the most significant, contributing 43.17% and 31.45%, respectively, and positive correlations (0.72 and 0.61, respectively) indicating their influence on vertical separation (Figure 3).

This analysis also indicated that the ordination reflects patterns associated with both species and sites (Figure 3). The most notable is that of the epiphytic species *Polyphlebium capillaceum*, which demonstrated a strong relationship with the SLA trait, distinguishing itself from the other species. Similarly, the terrestrial species *Parablechnum schiedeanum* and *Pteris orizabae* exhibited strong relationships with LDMC and LA, also setting themselves apart from the rest. In contrast, the epiphytic species *Vittaria graminifolia* was organized in the opposite direction of the LA and LDMC traits. These four species were exclusively found at the CLA and MAR sites, suggesting an association with these locations, while the remaining species, including those from NAT and KAN, did not exhibit clear ordination patterns.

### 2.3. Generalized Linear Mixed Model

The model indicated that random effects associated with differences among individuals and species carried a significantly greater weight in explaining variability (LA: R^2^c = 0.678; SLA: R^2^c = 0.162; LDMC: R^2^c = 0.036; VD: R^2^c = 0.298) compared to fixed effects (R^2^m = 0.000). Nevertheless, it remained evident that morphological traits are substantially influenced by site, habit, and their interactions, with CLA serving as the reference site. For the trait LA, the terrestrial habit exhibited a significant positive effect (*p* = 0.006), indicating that leaves of terrestrial ferns tend to be larger in size, particularly in CLA and MAR (Table 1; Supplementary Figure S1a). Meanwhile, this effect was significantly reduced in NAT (β = −0.75, *p* < 0.001) and KAN (β = −0.45, *p* = 0.005), as shown by the negative interaction terms. Regarding SLA, the site displayed a significant positive effect (*p* < 0.001), being highest in KAN (β = 0.17, *p* < 0.001) and significantly decreasing in the leaves of terrestrial species in MAR (β = −0.10, *p* = 0.017; Table 1, Supplementary Figure S1b).

For LDMC, site (*p* < 0.001) and its interaction with habit (*p* = 0.012) exhibited a significant positive effect. In this context, KAN and NAT recorded significantly lower values (β = −0.16, *p* < 0.001; β = −0.08, *p* = 0.003), whereas MAR showed higher values (β = 0.07, *p* < 0.001; Table 1, Supplementary Figure S1c). Additionally, interactions demonstrated a significant increase in KAN (β = 0.16, *p* = 0.012) and MAR (β = 0.11, *p* = 0.001) terrestrial leaves. Ultimately, terrestrial leaves displayed the highest VD (β = 0.72, *p* = 0.027), particularly at the MAR site (β = 0.07, *p* = 0.006; Table 1, Supplementary Figure S1d). Conversely, a significant decrease was noted in the terrestrial leaves of NAT and KAN (β = −0.43, *p* < 0.001; β = −0.20, *p* = 0.004).

## 3. Discussion

### 3.1. Microclimatic Variation

The higher values of canopy openness, radiation transmittance, and temperature in KAN and NAT result from their vegetation structure and conservation status [48] (Table 2). Human modifications to the sites have decreased vegetation cover and tree density, leading to more clearings and allowing for increased sunlight penetration [50,51]. This change has increased the total radiation transmittance and ultimately raised the understory temperature [52,53,54]. The reduced vegetation cover in these sites also leads to lower thermal buffering and evapotranspiration capacity [11,55], which explains why they exhibited low daily relative humidity values and a gradual decline over the two-year period (Figure 2). In addition, NAT and KAN showed the highest frequency of days exceeding 23 °C and below 70% (Supplementary Tables S1 and S2). These specific variations, although moderate individually, accumulate over time and significantly shape the site’s microclimate, generating thermal and hydric stress in the understory. Furthermore, days with temperatures exceeding average maximum temperatures [55] often coincide with particularly hot periods during which the average temperature for the region is exceeded (18 °C). This suggests that, during an already warm and stressful season for certain groups, such as ferns, the level of thermal stress intensifies in these sites. It has been documented that in ferns of the humid montane forests of this region, an average increase of 1 °C associated with changes in forest conditions can cause a decrease of up to 37–63% in their richness [27]. Moreover, the urban heat island effect caused by the proximity of these sites to urban areas may also contribute to the temperature rise, which is consistent with previous findings in forests in the area, showing that urban environments significantly raise air temperature due to reduced vegetation and high impermeable surface cover [46,49].

The CLA site, in contrast, exhibits a denser vegetation cover, featuring a forest structure indicative of a higher conservation status [48]. This condition resulted in reduced values of canopy openness and consequently lower levels of radiation transmittance and temperature (Figure 1 and Figure 2a). In this scenario, the site’s vegetation functions as a thermal buffer and a direct source of water vapor, helping to mitigate extreme temperature fluctuations and fostering more stable conditions within the forest [10,56,57]. This is further reflected in the fact that it exhibited the lowest daily fluctuations in temperature and relative humidity (Figure 2).

On the other hand, MAR had the lowest daily temperature and the highest daily relative humidity (Figure 1). In this case, altitude was the main factor determining microclimatic variation, surpassing the influence of vegetation structure and cover, as it is the only site located approximately 300 m higher than the others (Table 2). The lower temperature aligns with previous studies indicating a decrease in temperature with increasing altitude, with an average decrease of 0.5 °C for every 100 m of elevation [58,59]. Similarly, MAR’s higher altitude favored relative humidity retention, as the persistence of condensation increases in HMF as it approaches the cloud belt, typically found between 2000 and 3000 masl [60,61,62]. Furthermore, the drop in temperature with altitude leads to cooler air, which promotes moisture retention and condensation, thereby increasing water vapor presence in the environment [63,64].

### 3.2. Microclimatic Influence on Morphological Variation

The statistical model used indicates that species and individuals account for a considerable proportion of the total variability. However, significant differences related to site and habitat confirm their substantial influence on the morphological traits of the ferns in this study. This suggests that these are relevant variables capable of modulating the functional responses of this plant group, especially in urban environments.

Leaf area (LA) is a trait associated with ecological strategies regarding nutrient availability and resource utilization, as well as allometric factors such as plant size [65]. In this study, the leaves of terrestrial species were notably larger than those of epiphytes. This difference may arise from the more stressful conditions that epiphytic species typically face while growing in the tree canopy [66,67]. The increased solar exposure and reduced water availability tend to favor smaller leaves, which help conserve resources, lower water demand, and optimize water use efficiency [66,68,69], possibly explaining these results. A particularly strong positive effect of the terrestrial habit was observed in CLA and MAR, likely associated with the presence of *Pteris orizabae* and *Parablechnum schiedeanum*, two large terrestrial species found exclusively in these sites [69]. In contrast, the interaction effect between terrestrial habit and site was significantly lower in NAT and KAN, indicating that the difference in leaf area between terrestrial and epiphytic species was less pronounced. This reduction may be explained, conversely, by the absence of these large species [70], which considerably raise the mean leaf area in CLA and MAR. These findings suggest that species composition modulates the expression of habit effects at each site and further support the idea that species identity contributes substantially to the variability explained by the model.

Specific leaf area (SLA) indicates how plants allocate resources for leaf growth, reflecting their ability to capture light and photosynthesize [65,71]. Leaves from terrestrial and epiphytic species exhibited the highest values of SLA in CLA and KAN (Table 1). In the case of CLA, this suggests that individuals benefit from the dense canopy and use efficient photosynthetic mechanisms to maximize absorption in low-light conditions, which may indicate a significant adaptation of the associated ferns to thrive in umbrophilous environments. (e.g., *Asplenium miradorense* Liebm.) [25,69,70]. This is consistent with findings that showed that many epiphytic ferns adopt acquisitive strategies characterized by high SLA and low tissue density, particularly in humid, shaded microhabitats where maximizing light capture is advantageous [72,73,74].

Conversely, in KAN this result may relate to the presence of terrestrial disturbance indicator species, such as *Adiantopsis radiata* (L.) Fée, *Pteris pulchra* Schltdl. and Cham., and *Christella dentata* (Forssk.) Brownsey & Jermy [70]. These species have diverse functional response capacities, enabling them to capitalize on greater light availability, enhancing their photosynthetic capacity, and optimizing growth and development in disturbed environments [71,75]. This is particularly common in species of the Polypodiaceae family, which have been shown to have the ability to occupy more variable or disturbed environments, ranging from humid conditions to more exposed microhabitats [74].

Leaf dry matter content (LDMC) determines the physical resistance of leaves and their resource storage capacity [65,76]. In MAR, the highest LDMC values were found in the leaves of terrestrial and epiphytic species (Table 1, Supplementary Figure S1). This response is typically linked to stressful conditions and, in this case, can be attributed to low temperatures and abrupt decreases in relative humidity at the site (Figure 1 and Figure 2). Evidence suggests that as temperatures drop, plants tend to produce leaves with higher dry matter content as a mechanical support mechanism to endure cold conditions [77,78,79]. This occurs due to a greater number of cell layers, resulting in structurally denser leaves that are better equipped for heat retention, thanks to the high thermal capacitance provided by the water in these cells [80,81]. Research on ferns has linked increased lamellar thickness to higher LDMC in extreme conditions of greater altitude, which correspond to lower temperatures and increased aridity [31,37], so the situation observed in MAR aligns with this response.

Regarding the significant increase in dry matter in the leaves of terrestrial KAN species, this can also be attributed to stressful site conditions related, conversely, to higher solar radiation, elevated temperatures, and reduced water availability. This can be explained by the increased thickness of cell walls resulting from the compaction of mesophyll cells, which aids in water conservation under water-stress conditions [68,78,79]. This is linked to the high SLA values at this site, suggesting that greater light availability encourages disturbance indicator species. These species also exhibit a relatively robust structure that helps them endure challenging site conditions [68,80]. In contrast, epiphytic species in both CLA and KAN exhibited lower LDMC values, suggesting a different strategy. Epiphytic ferns may prioritize flexibility in water uptake because they are exposed to variable canopy microenvironments, with intermittent moisture sources [66,69,74]. So rather than investing in denser tissues, they may rely on reduced leaf area or increased water storage capacity.

Finally, the density of venation (VD) is often associated with facilitating water transport and thermal regulation in hot environments or those with limited water availability [62,81,82]. In ferns, it has been documented that vein density has a significant relationship with ambient humidity, decreasing as humidity increases [31]. In this study, the highest values of this trait were observed in the leaves of terrestrial species at the site with the highest relative humidity, MAR (Table 1, Supplementary Figure S1d). However, this trait is highly variable among species and is influenced significantly by phylogeny [65]. Thus, this result may be attributed to the influence of the large terrestrial species, *Pteris orizabae* and *Parablechnum schiedeanum*, which are specifically found at the sites with the highest relative humidity, CLA and MAR.

### 3.3. Morphological Differentiation and Tendency Towards Biotic Homogenization in Urban Forests

Two species exhibiting differential arrangement are *Polyphlebium capillaceum* and *Vittaria graminifolia*, both epiphytic ferns known as indicator species of natural forests, as they typically thrive in humid and shaded environments [27,29,82,83]. In particular, filmy ferns like *P. capillaceum* are sensitive to environmental changes due to their thin leaf structure and lack of cuticle, making them dependent on high relative humidity and shaded conditions [35,84,85]. These characteristics were evident through their corresponding morphological responses in the PCA (Figure 3), where *P. capillaceum* was observed to be strongly associated with the SLA trait, reflecting its thin and delicate leaves, while *V. graminifolia* appeared in the opposite direction, also demonstrating its small leaf size but a lower dry matter content.

Similarly, the terrestrial species *Parablechnum schiedeanum* and *Pteris orizabae* showed evident morphological responses, with a strong association with LDMC and LA traits (Figure 3), indicating a robust leaf structure and higher biomass investment. These species are commonly found in low-disturbance environments, where light, water, and nutrient availability are not limiting for their establishment [27,66,69]. Their ordination patterns in the PCA therefore suggest a strategy oriented towards structural persistence and competitive performance.

These four species (*P. capillaceum*, *V. graminifolia*, *P. schiedeanum*, and *P. orizabae*) were found only in CLA and MAR, the sites with the most mature forests [48,70]. By presenting a morphological response distinct from that of the other species (Figure 3), they indirectly reflect the microclimatic and structural conditions of these sites. This indicates that species sensitive to disturbance and changes in the microclimate are linked to forests with a higher degree of conservation [27,29]. These findings align with previous reports of other conservation indicator fern species at the CLA and MAR sites, such as the epiphytic fern species *Asplenium sphaerosporum* A.R.Sm. and *Didymoglossum reptans* (Sw.) C. Presl, as well as the terrestrial *Asplenium miradorense* Liebm. [70]. Furthermore, these distinct morphological patterns and their contribution to overall trait variability (Figure 3, Table 1) reflect the greater diversity and heterogeneity present in the best-preserved forests. This supports the idea that structurally complex environments promote a broader spectrum of functional strategies, both acquisitive and conservative, especially in ferns [74].

In contrast, the leaves of the NAT and KAN sites showed more clustered positions in the PCA space, indicating less variation in morphological traits across species (Figure 3). This pattern likely reflects the environmental filtering imposed by more open and disturbed forest structures, which tend to select for species with similar ecological strategies, such as tolerance to increased light and temperature, and reduced humidity, and displacing more sensitive ones, such as the four aforementioned species [15,18]. The frequent presence of species identified as disturbance indicators, such as the terrestrials *Adiantopsis radiata* (L.) Fée, *Pteris pulchra* Schltdl. & Cham., and *Christella dentata* (Forssk.) Brownsey & Jermy, common in NAT and KAN but absent in CLA and MAR, reinforces this interpretation [70].

Although variation among species is still present, the reduced morphological diversity and limited ordination patterns in NAT and KAN suggest an early stage of biotic homogenization, where environmental stress filters out species with narrower ecological requirements and favors the persistence of generalist and tolerant taxa. This aligns with observations in other urban green areas in tropical regions worldwide with similar environmental conditions, where the flora underwent homogenization due to environmental filters imposed by urban expansion, resulting in a decline in species richness and diversity [16,86,87,88].

## 4. Materials and Methods

### 4.1. Study Sites

**Figure 4:**
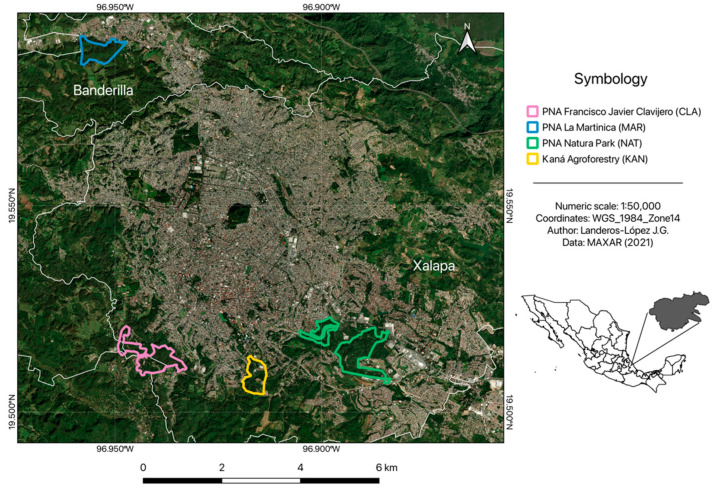
Spatial locations of the four selected urban forest sites in the Xalapa–Banderilla conurbation area, Veracruz State, Mexico.

The study was conducted in the Xalapa–Banderilla conurbation area, located in Veracruz State, Mexico, between the coordinates 19°29′ and 19°36′ N latitude and 96°48′ and 96°58′ W longitude, with an altitude ranging from 1120 to 1700 masl [89] (Figure 4). The region’s climate is semi-warm–humid, with an average temperature of 18 °C and an average annual rainfall of 1500 mm [90]. The most prevalent vegetation type in the area is the humid montane forest (HMF), although other communities, such as oak, pine, and low deciduous forests, are also present [91,92]. Currently, this area has a combined population of over 500,000 inhabitants, making it one of the fastest-growing urban areas in the state [93,94].

The four urban forest sites selected for this study include Natura Park (NAT), part of the Protected Natural Area (PNA) “El Tejar Garnica”; Kaná Agroforest (KAN), affiliated with the Campus for Culture, Arts, and Sports of the University of Veracruz; the Francisco Javier Clavijero PNA (CLA), situated in a polygon managed by the Ministry of Environment of Veracruz; and the PNA “La Martinica” (MAR; Figure 4). These forests exhibit remnants of HMF, yet they differ in structure and plant composition due to varying processes of anthropic modification and the influence of the urban environment [48] (Table 2). For instance, NAT and KAN are fully embedded in the urban sprawl, leading to total or partial isolation caused by avenues, shopping centers, and other urban developments. In these areas, secondary tree species dominate (e.g., *Dendropanax arboreus* (L.), *Citharexylum caudatum* L., *Piper amalago* L., *Vachellia pennatula* (Schltdl. & Cham.) Seigler & Ebinger), while primary HMF species are observed to a lesser extent. Conversely, CLA and MAR are located on the periphery, in less developed regions, exhibiting a tree species composition more similar to mature forests (*Liquidambar styraciflua* L., *Quercus* L. spp.) [48] (Figure 4).

### 4.2. Data Collection

For the evaluation of microclimatic conditions, three digital sensors (Track-It™ RFID Dataloggers) were placed on tree trunks and branches at a height of 2 m per site to record temperature and relative humidity every hour from August 2021 to August 2023. In the case of MAR and KAN, data loss occurred during March and April 2022 due to sensor malfunction. As a result, the total number of days recorded per site was as follows: CLA—674, MAR—621, NAT—699, and KAN—642. During the same period, one hemispherical photograph was taken at each of the five sample plots established in a previous study [48] to describe the light environment. The photographs were captured at a height of 1 to 1.5 m in the central area of each plot to minimize the influence of the edges. In total, five photographs were obtained per site, which were subsequently analyzed using the Gap Light Analyzer application [95] to determine canopy openness and solar radiation transmittance.

For the evaluation of morphological traits in ferns, individuals were collected in the same established plots where the structure and composition of the woody vegetation, as well as the diversity of ferns, have been characterized [48,70]. Since the sites exhibit heterogeneous communities in terms of species count [70], this study considered the most frequent terrestrial and epiphytic species that represent at least 30% of the total fern richness at each site [70]. Therefore, a total of 13 species were selected, distributed as follows: 10 at the CLA site, 11 at MAR, and 7 both at NAT and KAN (Table 3).

**Figure 5:**
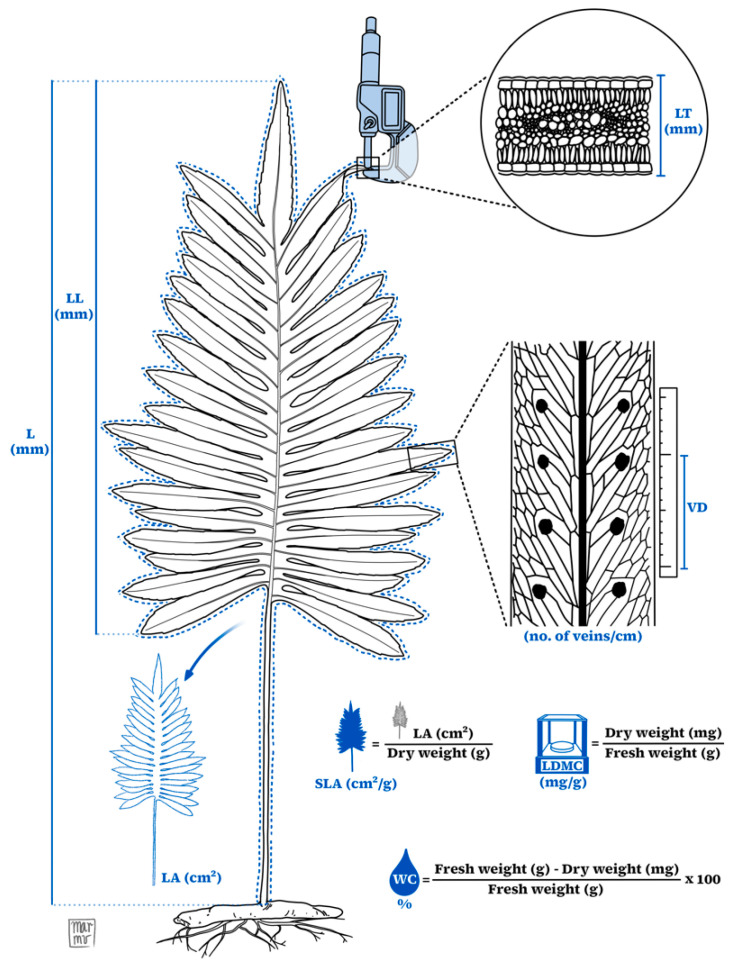
Illustrative scheme for measuring the eight morphological traits of a fern leaf. L: total length, LL: lamina length, LA: leaf area, SLA: specific leaf area, LT: leaf thickness, LDMC: leaf dry matter content, WC: water content, VD: vein density. Concept by Landeros-López, J.G. Illustrated by Muñoz-Velázquez, M.

Thirty fronds per species were obtained by sampling a variable number of individuals according to their availability, with each individual contributing a different number of fronds. Individuals considered healthy and mature were collected with their entire rhizome and allowed to rehydrate for 24 h in resealable plastic bags with the rhizome submerged in water. Subsequently, the fronds were transported to the laboratory, where eight morphological traits were measured following Pérez-Harguindeguy et al. (2013) [65]: total length (L), lamina length (LL), leaf area (LA), specific leaf area (SLA), leaf thickness (LT), leaf dry matter content (LDMC), water content (WC), and vein density (VD) (Figure 5). For compound-leaved fern species, we included the petiole area as part of the total leaf area (LA), as it represents a significant portion of the leaf structure and contributes to its functional surface. This approach is consistent with established protocols, which indicate that including the petiole is valid depending on the study context [65].

### 4.3. Data Analysis

Daily means of temperature and relative humidity, and overall means of canopy openness and radiation transmittance, were evaluated using the Shapiro–Wilk normality test. Since temperature and relative humidity data did not follow a normal distribution (Shapiro–Wilk: *p* < 0.0001), their means were compared using the non-parametric Kruskal–Wallis test. In contrast, canopy openness and radiation transmittance data were normally distributed (Shapiro–Wilk: *p* > 0.05), so their means were analyzed using one-way ANOVA. Post hoc comparisons were performed to identify significant differences between groups. For ANOVA, Tukey’s Honest Significant Difference (HSD) test was applied using the TukeyHSD() function, which includes built-in correction for multiple comparisons. For Kruskal–Wallis tests, pairwise comparisons were run with pairwise.wilcox.test() using Holm-adjusted *p*-values (p.adjust.method = “holm”). Letters indicating group differences were added to the graphs based on these post hoc results.

Additionally, time series were generated to visualize daily variation in temperature and relative humidity. These series were constructed by using daily means for each variable across the four study sites and organizing them chronologically. To facilitate interpretation, we included reference lines representing extreme thresholds of temperature and humidity reported for HMF in the region [27,49]. Summary tables indicating the number of days in which values exceeded these thresholds are provided to support the visual interpretation of the time series (see Supplementary Tables S1 and S2).

The morphological variables were also evaluated using the Shapiro–Wilk normality test. Since the data did not follow a normal distribution (Shapiro–Wilk: *p* < 0.0001), a Spearman correlation analysis was performed for non-parametric data. This allowed us to eliminate highly correlated variables (ρ > 0.80) that provided redundant information. Spearman correlation analysis revealed strong correlations among several morphological traits, indicating potential redundancy (See Supplementary Figure S2). In these cases, highly correlated variables may provide overlapping information, which can distort multivariate analyses or inflate the importance of certain trait dimensions. Therefore, to reduce redundancy and improve interpretability, total length (L) and lamina length (LL) were excluded because of their high correlation with leaf area (LA; r > 0.90). Leaf thickness (LT) was also removed because of its high negative correlation with specific leaf area (SLA; r = −0.81). Similarly, leaf dry matter content (LDMC) and water content (WC) exhibited a strong negative correlation (r = −0.98); LDMC was retained due to its greater functional importance. Therefore, the traits retained for further analysis included LA, SLA, LDMC and vein density (VD).

Using the selected variables, a principal component analysis (PCA) was performed to observe the ordination of traits according to species and sites. Prior to the analysis, all variables were standardized using z-score standardization with the scale() function from base R, which centers and scales each variable to have a mean of zero and a standard deviation of one. Finally, a generalized linear mixed model (GLMM) was fitted to evaluate the effects of site, habit (terrestrial or epiphytic), and their interaction on morphological traits, considering a random effect of the leaves of each individual nested within the species to account for the individual effect of species richness at each site. The model employed a Gaussian distribution with a logarithmic link. This choice was made because the morphological trait data were strictly positive and continuous, and the residuals deviated from normality and homoscedasticity. The log link is recommended in such cases to stabilize variance and improve model performance [96,97]. In addition, following the recommendations of previous studies, the transformation was applied within the model framework rather than to the raw data [98]. Therefore, its final form was as follows: glmer((X) ~ Site + Habit + Site:Habit + (1 | Species/Individual), data = Data, family = gaussian(link = “log”)), where X corresponds to each trait evaluated. The fit of the variables in the model was evaluated using pseudo-conditional and marginal R^2^ values [99]. The corresponding analyses and graphs were executed using the statistical programs RStudio version 2024.09.1 (with the packages nortest, psych, Hmisc, FactoMineR, factoextra, stats, ggplot2, tidyverse, dplyr, grid, lme4, and lubridate) and JAMOVI version 2.3.28 [100,101]. Normality tests (Shapiro–Wilk), as well as ANOVA and Kruskal–Wallis analyses, were performed using the functions from the base R package.

## 5. Conclusions

In the studied urban forests of central Veracruz, ferns exhibited morphological variation in response to microclimatic and structural changes. Despite the variation caused by species and individuals, both site and habitat were determining factors for the evaluated morphological traits. Generally, responses were linked to microclimatic variation at the four sites, where traits related to resource acquisition influenced morphological variation. This was evident through the optimization of light capture and photosynthetic efficiency in response to changes in light availability, as well as water and biomass conservation due to water limitations. Overall, this provides evidence of the group’s ability to respond even to minimal environmental variations, demonstrating that, like other plant groups, they possess mechanisms that allow them to adapt to different environments. This contributes to their persistence in diverse habitats and makes them ideal subjects for studying adaptation and ecological transformation processes.

When analyzing growth habits, terrestrial ferns tended to have larger leaves, a greater specific leaf area, and a higher dry matter content in wetter and shaded sites—traits consistent with better resource acquisition under favorable conditions. In contrast, in drier and more open environments, these species exhibited smaller leaves and lower SLA, likely adapting to water stress. Epiphytic ferns, on the other hand, generally displayed smaller, thicker leaves, with lower SLA and LDMC across all sites, where slight variations suggested a more conservative strategy adapted to limited water access and canopy exposure. Meanwhile, vein density was more closely associated with a taxonomic response. Overall, these patterns indicate that terrestrial species exhibit greater morphological plasticity across environmental gradients, while epiphytes maintain more stable trait expressions, possibly due to greater physiological constraints associated with their habit.

Microclimatic variations in central Veracruz’s urban forests primarily result from structural modifications, which appear to favor generalist species while displacing native specialists. This leads to a predominance of similar morphological traits and reduced diversity, potentially limiting the ecological functionality of these urban ecosystems. The principal component analysis (PCA) performed provides initial evidence that most of the studied fern species share similar morphological traits, regardless of the site’s degree of urbanization, highlighting a clear trend toward biotic homogenization. Hence, this study also serves as a reminder of the ongoing fundamental role of old-growth forests in biodiversity conservation and ecosystem functioning [102]. While urban forests are often regarded as biodiversity refuges and vital sites for microclimate maintenance, their proximity to urban environments and constant anthropogenic modifications are key factors that make them potential refuges for generalist and tolerant species [4,103].

Finally, this study highlights relevant trends in a group that has been explored little in urban and functional contexts, emphasizing the impact of human modifications on their biodiversity and functionality within urban ecosystems. We acknowledge the limitations of working with this group due to the challenges of measuring morphological traits in ferns; however, these limitations do not diminish the value of the observed patterns, which contribute to a better understanding of their ecological responses. For future related research, we suggest considering the inclusion of species-specific abundance data, as this would allow for a more robust assessment of functional diversity metrics and the processes associated with homogenization.

## Figures and Tables

**Table 1:** Generalized linear mixed models for the evaluation of morphological traits of terrestrial and epiphytic ferns present at the four study sites. Estimates, 95% confidence intervals (CIs), t-values, and *p*-values are shown for fixed effects. Marginal and conditional R^2^ values (R^2^m/R^2^c) indicate the proportion of variance explained by fixed effects alone and by both fixed and random effects, respectively. LA: leaf area, SLA: specific leaf area, LDMC: leaf dry matter content, VD: vein density; CLA: Francisco Javier Clavijero; MAR: La Martinica; NAT: Natura Park; KAN: Kaná Agroforestry.

Trait	Predictor	Estimate (β)	CI (95%)	t	*p*	R^2^ m/R^2^ c
LA	(Intercept)	3.59	2.55–4.63	6.756	<0.001	0.000/0.678
	Site KAN	0.03	−0.16–0.21	0.275	0.783	
	Site MAR	−0.05	−0.17–0.07	−0.781	0.435	
	Site NAT	0.17	0.01–0.33	2.142	0.032	
	Terrestrial Habit	2.15	0.62–3.69	2.748	0.006	
	SiteKAN × TerrestrialHabit	−0.49	−0.83–−0.15	−2.798	0.005	
	SiteMAR × TerrestrialHabit	−0.12	0.34–0.09	−1.158	0.247	
	SiteNAT × TerrestrialHabit	−0.75	−1.05–−0.45	−4.875	<0.001	
SLA	(Intercept)	5.13	4.80–5.46	30.661	<0.001	0.000/0.162
	Site KAN	0.17	0.09–0.25	4.027	<0.001	
	Site MAR	−0.03	−0.07–0.00	−1.823	0.069	
	Site NAT	0.02	−0.07–0.11	0.417	0.677	
	TerrestrialHabit	0.18	−0.31–0.67	0.727	0.467	
	SiteKAN × TerrestrialHabit	0.07	−0.08–0.23	−0.950	0.342	
	SiteMAR × TerrestrialHabit	−0.10	−0.19–−0.02	2.401	0.017	
	SiteNAT × TerrestrialHabit	0.08	−0.07–0.23	1.030	0.303	
LDMC	(Intercept)	5.59	5.44–5.73	75.152	<0.001	0.000/0.036
	Site KAN	−0.16	−0.23–−0.10	−4.953	<0.001	
	Site MAR	0.07	0.03–0.11	3.818	<0.001	
	Site NAT	−0.08	−0.13–−0.03	−3.006	0.003	
	TerrestrialHabit	0.07	−0.15–0.29	0.634	0.526	
	SiteKAN × TerrestrialHabit	0.16	0.04–0.29	2.515	0.012	
	SiteMAR × TerrestrialHabit	0.11	0.05–0.18	3.422	0.001	
	SiteNAT × TerrestrialHabit	−0.02	−0.13–0.09	−0.281	0.779	
VD	(Intercept)	1.68	1.25–2.11	7.649	<0.001	0.050/0.298
	Site KAN	0.03	−0.04–0.10	0.810	0.418	
	Site MAR	0.07	0.02–0.12	2.746	0.006	
	Site NAT	0.07	0.00–0.13	2.048	0.041	
	TerrestrialHabit	0.72	0.08–1.35	2.222	0.027	
	SiteKAN × TerrestrialHabit	−0.20	−0.33–−0.06	−2.884	0.004	
	SiteMAR × TerrestrialHabit	−0.14	−0.22–−0.05	−3.137	0.002	
	SiteNAT × TerrestrialHabit	−0.43	−0.55–−0.31	−7.125	<0.001	

**Table 2:** Description of the vegetation structure and composition of the four urban forests comprising the study sites. DBH: diameter at breast height; PNA: protected natural area [48].

Urban Forest	Area (ha)	Altitude (masl)	Basal Area (m^2^/ha)	DBH (cm)	Height (m)	Density (tree/ha)	State of Maturity of the Vegetation
PNA Francisco Javier Clavijero (CLA)	22.06	1362	3.3 ± 1.14	19.08 ± 21.89	13.48 ± 7.19	32 ± 46	Mature forest with fragments of secondary forest
PNA La Martinica (MAR)	52.30	1599	1.6 ± 0.72	13.57 ± 14.66	14.20 ± 6.80	49 ± 78	Mature forest with fragments of secondary forest
PNA Natura Park (NAT)	80	1320	1.6 ± 0.40	14.92 ± 13.38	11.20 ± 5.50	46 ± 75	Secondary forest, with few remaining trees of mature forest
Kaná Agroforestry (KAN)	5	1366	1.9 ± 0.56	14.86 ± 16.27	9.88 ± 5.73	37 ± 35	Secondary forest with floristic elements of mature forest

**Table 3:** This study selected species of terrestrial and epiphytic ferns due to their abundance. T: terrestrial, E: epiphytic. CLA: Francisco Javier Clavijero, MAR: La Martinica, NAT: Natura Park, KAN: Kaná Agroforest. The letter X indicates presence at the site.

Family	Species	Habit	CLA	MAR	NAT	KAN
Pteridaceae	*Adiantopsis radiata* (L.) Fée	T			X	
Aspleniaceae	*Asplenium miradorense* Liebm.	T	X	X		
Blechnaceae	*Blechnum appendiculatum* Willd.	T	X	X	X	X
Thelypteridaceae	*Christella dentata* (Forssk.) Brownsey & Jermy	T			X	X
Blechnaceae	*Parablechnum schiedeanum* (Schltdl. ex C. Presl) Gasper & Salino	T		X		
Polypodiaceae	*Phlebodium pseudoaureum* (Cav.) Lellinger	E	X	X	X	X
Polypodiaceae	*Pleopeltis crassinervata* (Fée) T. Moore	E	X	X	X	X
Polypodiaceae	*Pleopeltis furfuracea* (Schltdl. & Cham.) A. R. Sm. & Tejero	E	X	X	X	X
Polypodiaceae	*Pleopeltis plebeia* (Schltdl. & Cham.) A. R. Sm. & Tejero	E	X	X	X	X
Hymenophyllaceae	*Polyphlebium capillaceum* (L.) Ebihara & Dubuisson	E	X	X		
Polypodiaceae	*Polypodium conterminans* Liebm.	E	X	X		
Pteridaceae	*Pteris orizabae Mart. & Galeotti*	T	X	X		
Pteridaceae	*Vittaria graminifolia* Kaulf.	E	X	X		

## Data Availability

The original contributions presented in this study are included in the article/supplementary material. Further inquiries can be directed to the corresponding author.

## Supplementary Materials

The following supporting information can be downloaded at https://www.mdpi.com/article/10.3390/plants14111732/s1.
