# Supplementary material for: Influence of Microclimatic Variations on Morphological Traits of Ferns in Urban Forests of Central Veracruz, Mexico
Source: Plants (Basel). 2025 Jun 5;14(11):1732. doi: 10.3390/plants14111732 (PMC12157192; doi:10.3390/plants14111732)
Supplement: Supplementary file 1 [file plants-14-01732-s001.zip › plants-3611841-supplementary.pdf]

Supplementary Material

Figure S1. Morphological traits of terrestrial and epiphytic ferns present at the four study sites. (a): Leaf area; (b): Specific leaf area; (c): Leaf dry matter content; (d): Vein density. A blue asterisk indicates a significant difference ($p < 0.05$) of the site compared to the CLA reference site; a red asterisk indicates a significant difference ($p < 0.05$) of the site:habit interaction compared to the CLA reference site.

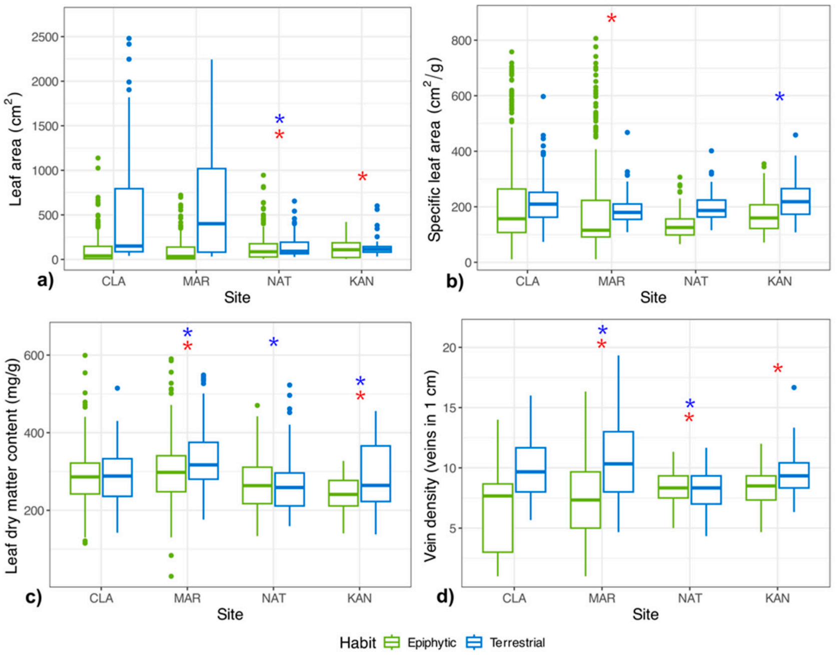

Figure S2. Spearman correlation matrix of the eight measured morphological traits of ferns at the four study sites.

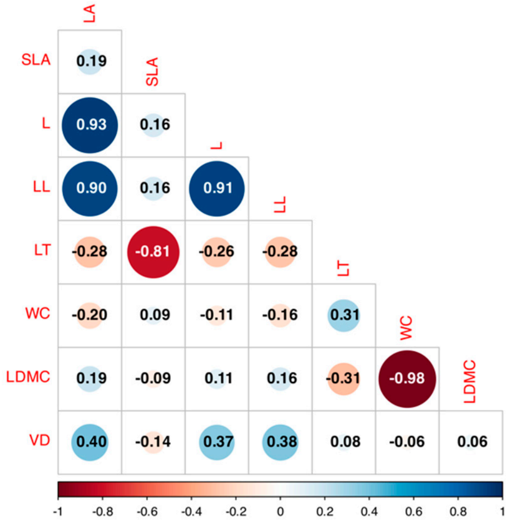

Table S1. Number of days per site of daily temperature values exceeding predefined thresholds in the time series.

Site	< 16 °C	Percentage of recorded days (%)	> 17.10 °C	Percentage of recorded days (%)	> 23 °C	Percentage of recorded days (%)
CLA	217 days	32.19	368 days	54.59	2 days	0.29
MAR	280 days	45.08	189 days	30.43	0 days	0
NAT	158 days	22.60	446 days	63.80	16 days	2.28
KAN	158 days	24.61	409 days	63.70	10 days	1.71

Table S2. Number of days per site of daily relative humidity values exceeding predefined thresholds in the time series.

Site	< 70%	Percentage of recorded days (%)	> 90%	Percentage of recorded days (%)	> 94.41%	Percentage of recorded days (%)
CLA	2 days	0.29	536 days	79.52	446 days	71.81
MAR	8 days	1.28	525 days	84.54	404 days	59.94
NAT	4 days	0.57	563 days	80.54	469 days	67.09
KAN	11 days	1.71	519 days	80.84	437 days	68.06
